# Molecular epidemiology, prenatal exposure and prevention of cancer

**DOI:** 10.1186/1476-069X-10-S1-S5

**Published:** 2011-04-05

**Authors:** Frederica Perera

**Affiliations:** 1 Mailman School of Public Health, Columbia University, 100 Haven Avenue, Tower 3, #25F, New York, NY 10032, USA

## Abstract

Molecular Epidemiology was originally conceived as a preventive approach, providing a valuable tool for investigating risk factors for cancer in vulnerable populations. Biomarkers can be used as early indicators of risk for preventative purposes and risk assessment. The present contribution mainly refers to *in utero* exposures to carcinogens, since humans are especially vulnerable during fetal development. Environmental exposures *in utero* can increase risks for both childhood and adult cancers; their interactions with genetic and nutritional susceptibility factors may further increase risk. Thus, the early developmental period represents an important window for cancer prevention.

## Introduction

Lorenzo Tomatis was an internationally recognized pioneer and leader in cancer prevention. The following remarks are made in honor of Lorenzo and his lifelong work. Molecular Epidemiology was conceived as a preventive approach, providing a valuable tool for investigating risk factors for cancer in vulnerable populations. Biomarkers can be used as early indicators of risk for preventative purposes and risk assessment. Humans are especially vulnerable during fetal development. Environmental exposures *in utero* can increase risks for both childhood and adult cancers; their interactions with genetic and nutritional susceptibility factors may further increase risk. Thus, the early developmental period represents an important window for cancer prevention.

## Background on fetal susceptibility

The fetus is generally at greater risk than the adult from toxic and carcinogenic environmental chemicals and other agents. A number of factors are involved: the high rates of absorption and retention of chemicals by the fetus, inability of the fetal liver to metabolize and detoxify toxins efficiently, the immaturity of fetal mechanisms of DNA repair and immune response, and the high rate of cell proliferation. In addition to directly damaging fetal cells and molecular/genetic targets, environmental chemicals can interfere with fetal development by disrupting the growth of the placenta and affecting the transplacental transfer of nutrients to the fetus. Thus, there is an increased likelihood that exposure to an environmental toxicant during the fetal period will result in procarcinogenic damage in the form of DNA adducts and mutation as well as epigenetic changes [1]. Such procarcinogenic damage acquired during the fetal period will likely persist through many more cell divisions than those acquired later in life. There is therefore, a greater opportunity for cancer and other chronic diseases to develop when the causative exposure is experienced *in utero.*

The importance of the fetal period is underscored by the growing number of environmental exposures that are known or suspected of increasing risk of childhood and adult cancer when the exposure occurs during the fetal period. These include air pollutants, such as polycyclic aromatic hydrocarbons (PAHs), tobacco smoke, pesticides, solvents, and radiation [2-7]. Fetal exposures to endocrine disruptors have also been linked to diverse health effects in animal studies (and to a limited extent in humans), including cancers, immune disorders, and obesity [8]. Thus, the fetal period represents an important opportunity for the prevention of cancer and other diseases.

## Results from recent molecular epidemiologic studies

Our studies at the Center for Children’s Environmental Health (CCCEH) relating various biologic markers to air pollutant exposures have highlighted the susceptibility of the fetus. In the New York City -based “Mothers and Newborns Cohort Study”, pregnant mothers wore personal air monitors to measure their exposure to PAHs during pregnancy [9]. PAHs are widespread carcinogens, resulting from combustion of fossil fuels and other organic material. PAH-DNA adducts have been associated with cancer, both experimentally and in humans. The level of prenatal maternal urinary PAH metabolites has been measured as well as PAH-DNA adducts in cord and maternal blood. Although the estimated dose of PAH to the fetus is about 10 times lower than to the mother, maternal and cord white blood cells had equivalent levels of PAH-DNA adducts [10]. This finding supports the increased susceptibility of the fetus to procarcinogenic DNA damage by these common environmental carcinogens [11].

In the same cohort study, lymphocytes were examined in cord blood using whole-chromosome painting and fluorescent in situ hybridization (FISH). High prenatal PAH exposure was associated with a higher frequency of stable chromosomal aberrations in cord blood [12,13]. These results showed a link between transplacental exposure to PAHs and genetic damage which may potentially increase risk of cancer. We are currently investigating the relationship between exposure, chromosomal aberrations and the TEL-AML1 preleukemic alteration.

My colleagues and I are evaluating the effects of prenatal exposures to PAHs and other environmental toxicants on epigenetic programming, specifically altered DNA methylation. DNA methylation is a covalent modification at cytosine residues of DNA, often occurring in promoter regions and inhibiting transcription [14]. DNA methylation is believed to play a role in tumor formation. One example is the hypermethylation of tumor suppressor genes leading to a loss of expression [15] and the global hypomethylation seen in lung cancer [16]. PAHs have been shown to alter DNA methylation patterns in various types of tumor cells. The fetus is particularly susceptible to exposures that can alter epigenetic markers because these patterns, which are generally heritable through successive cell divisions, are largely cleared and then re-established during early fetal development [17].

In our NYC cohort study, we have found that global DNA methylation levels in cord blood white blood cells decreased with increasing prenatal PAH exposure [Herbstman et al. 2010 in prep.]. Interestingly, we also found that global DNA methylation levels in cord blood were positively correlated with levels of PAH-DNA adducts in cord blood; thus adducts do not appear to mediate or modify the inverse association between PAH exposure and DNA methylation levels. These results indicate that the fetal epigenome is vulnerable to PAH exposure and that epigenetic dysregulation in the developing fetus due to PAH exposure does not directly involve PAH-DNA adduct formation. The results are consistent with experimental evidence that PAH-DNA adducts form preferentially at methylated DNA sites and that DNA methylation may make DNA more reactive and, therefore, more vulnerable to genetic damage by PAHs and other chemicals [18].

Regarding persistence of epigenetic changes due to prenatal exposure, comparison of methylation levels in cord blood and blood collected at age 3 from the same children showed that methylation levels did not vary significantly over time. This suggests that PAH-related epigenetic perturbations are relatively stable over this period and could contribute to a wide range of health effects throughout childhood and possibly later in life.

One of the disadvantages of the studies described above is that we measured overall DNA methylation levels, not the methylation status of specific genes. We are now examining the effects of prenatal PAH exposure on DNA methylation of specific genes. In a pilot study, our colleagues at the University of Cincinnati and we identified genes with differential methylation levels in cord white blood cells between children with low prenatal PAH exposure and high prenatal PAH exposure [19]. We are currently using high throughput genome wide arrays in our birth cohorts in NYC and Poland to identify additional genes whose methylation patterns are vulnerable to disease-inducing perturbation by PAHs.

## Conclusion

Our studies provide molecular and genetic evidence of widespread exposure to PAHs, increased fetal susceptibility, and links between *in utero* exposure and biomarkers of potentially increased cancer risk, such as adducts and chromosomal aberrations. We have also found that prenatal PAH exposure affects epigenetic patterning by altering DNA methylation. Epigenetic alterations have been shown to play a role in cancer, and have also been found to be associated with a wide range of other diseases. Thus, increased fetal susceptibility to environmental contaminants can contribute to molecular alterations, quantifiable as biomarkers, which potentially increased risk of cancer and multiple diseases that may present across the life course. Measuring biomarkers associated with fetal exposure can therefore inform risk assessment and early intervention programs aimed at preventing cancer and other environmentally-related diseases.

## Competing interests

The author declare no competing financial or non-financial interests.
